# An Investigation of the Sol-Gel Transition of Chitosan Lactate and Chitosan Chloride Solutions via Rheological and NMR Studies

**DOI:** 10.3390/gels8100670

**Published:** 2022-10-19

**Authors:** Katarzyna Pieklarz, Jacek Jenczyk, Zofia Modrzejewska, Piotr Owczarz, Stefan Jurga

**Affiliations:** 1 Department of Environmental Engineering, Faculty of Process and Environmental Engineering, Lodz University of Technology, 93-005 Lodz, Poland; 2 NanoBioMedical Centre, Adam Mickiewicz University, 61-614 Poznan, Poland; 3 Department of Chemical Engineering, Faculty of Process and Environmental Engineering, Lodz University of Technology, 93-005 Lodz, Poland

**Keywords:** biopolymers, chitosan, thermosensitive hydrogel, sol-gel transition, gelation point, rheology, NMR

## Abstract

In recent years, intensive research has been carried out on the use of hydrogels obtained from natural polymers, mainly chitosan. These products are increasingly replacing solutions based on synthetic materials in medicine. This publication presents the results of studies on the sol-gel transition of chitosan solutions as the base material for the preparation of thermosensitive hydrogels for potential applications in tissue engineering. The measurements were carried out for systems consisting of chitosan lactate and chitosan chloride solutions using β-glycerol phosphate disodium salt pentahydrate and uridine 5′-monophosphate disodium salt as the cross-linking agents. The sol-gel transition point of the solutions was determined based on the rheological measurements in the cone-plate configuration of the rotational rheometer and experiments performed using the method of nuclear magnetic resonance. The obtained results showed a significant influence of the cross-linking agent on the course of the sol-gel transition of chitosan salt solutions, and the systems that consisted of chitosan lactate seemed to be especially interesting for biomedical applications.

## 1. Introduction

The development of modern polymer materials, both for general use and special purposes, is increasingly based on the use of reproducible natural polymers, such as cellulose, starch, lignin, chitin, chitosan, or alginates. These valuable raw materials are obtained from biomass as well as fauna, especially marine fauna. The requirements that innovative biomaterials have to meet, apart from those related to their functional properties, are also associated with the possibility of their ecological utilization [1,2,3].

Among the many types of polymer products, much attention is currently being paid to hydrogels, which are spatially cross-linked materials with a three-dimensional structure capable of swelling in water or biological fluids [4,5].

The polymer chains that build the hydrogel network can be linked with chemical bonds, or their structure can be maintained with molecular bonds, additional ionic forces, hydrogen bonds, or hydrophobic interactions. When the hydrogel network is formed using chains connected with chemical bonds, the hydrogels reach an equilibrium swelling state and are called chemical gels. On the other hand, when non-covalent interactions occur, hydrogels are called physical [6,7,8].

Additionally, hydrogels can be divided into two categories: the so-called conventional systems composed of loosely connected hydrophilic, mostly non-ionic polymers with a high degree of swelling in water without dissolution, and hydrogels sensitive to the stimuli of the local environment, such as pH, temperature, electric potential, magnetic field, or light [5].

In recent years, there has been considerable progress in the use of hydrogels in medical science. These solutions are mainly used as dressing materials, carriers for the controlled release of drugs, surgical implants, biosensors, and to produce contact lenses [9,10,11].

Nevertheless, one of the key factors determining the suitability of hydrogels for biomedical use is ensuring the full biocompatibility of the material with the tissues of the human body. For this reason, particular attention is paid to systems based on chitosan, which is a deacetylated chitin derivative; this polysaccharide is insoluble in water but has good solubility in organic or inorganic acids, forming salts with solvent ions [12]. In an acidic environment, the hydrophobic properties of the polymer change to hydrophilic, leading to the formation of a colloidal system, which indicates the possibility of a sol-gel transition.

Chitosan hydrogels can be obtained by increasing the polymer concentration, changing the pH of the solution, or raising the temperature [13,14,15,16]. The last of these options leads to a change in the structure of the polymer chain and the formation of interactions between the functional groups of the chitosan chains after exceeding the lower critical solution temperature (LCST). The location of the phase transition point depends on the type of solvent used and the presence of substances affecting the pH of the solution.

Chitosan (CH), due to its favorable biological properties [17,18,19] and ability to create various morphological structures, such as microgranules, sponges, membranes, films, hydrogels, and fibers [20,21,22], is widely studied for its applications in tissue engineering, ranging from the development of functional substitutes for human skin, through to solutions enabling the regeneration of bones, cartilage, and nerves [23,24,25,26,27,28]. The possibility of using chitosan as a carrier for pharmacological preparations and the treatment of hypercholesterolaemia is also interesting [29,30,31].

The aim of this study was to create low-concentration colloidal chitosan solutions that demonstrate the ability to undergo a sol-gel change induced by temperature increases and to describe this transition by determining the gelation point. These systems are interesting as injectable scaffolds in tissue engineering.

Systems obtained from chitosan lactate and chitosan chloride solutions with the use of β-glycerol phosphate disodium salt pentahydrate (β-GP) or uridine 5′-monophosphate disodium salt (UMP) [32,33] as the cross-linking agents were investigated. Rheological measurements were carried out to define the sol-gel transition point of the analyzed chitosan systems, and their outcomes were compared with the results of experiments performed with nuclear magnetic resonance (NMR).

## 2. Results and Discussion

### 2.1. Non-Isothermal Sol-Gel Transition Process

**Figure 1:**
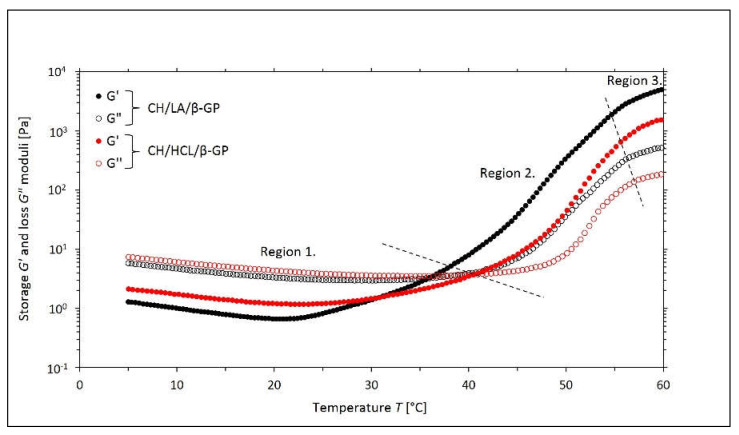
The experimental curves of the changes in the values of the storage *G*′ and loss *G*″ moduli as a function of the temperature *T* obtained for the chitosan lactate solutions (the CH/LA/β-GP system) and chitosan chloride (the CH/HCL/β-GP system) with β-glycerol phosphate disodium salt pentahydrate (β-GP).

**Figure 2:**
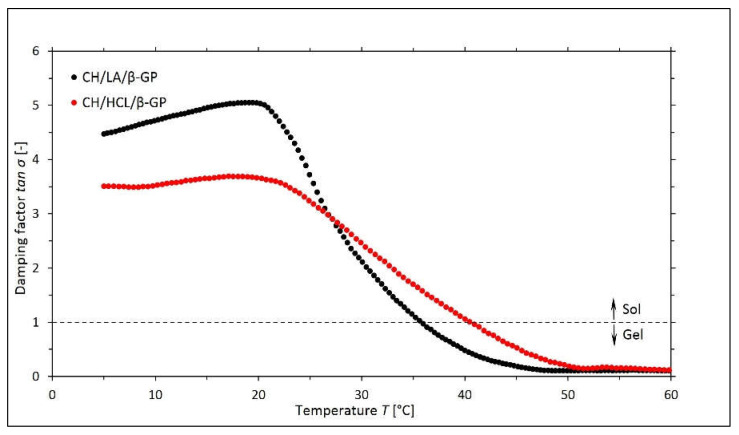
The experimental curves of the changes in the values of the damping factor tan σ *= G*″/*G*′ as a function of the temperature *T* obtained for the chitosan lactate solutions (the CH/LA/β-GP system) and chitosan chloride (the CH/HCL/β-GP system) with β-glycerol phosphate disodium salt pentahydrate (β-GP).

**Figure 3:**
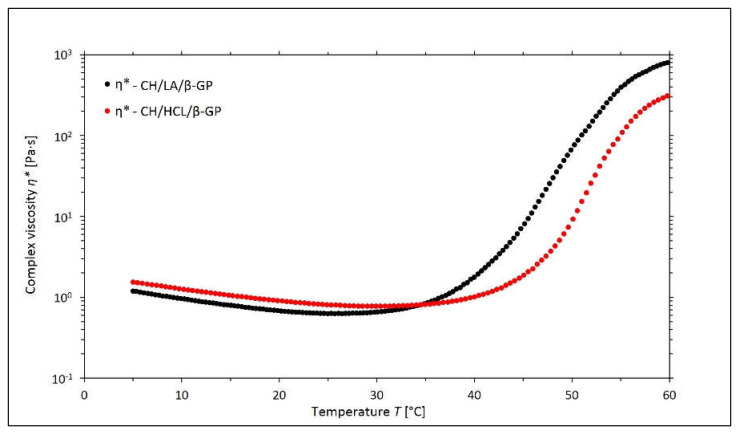
The experimental curves of the changes in the values of the complex viscosity η* as a function of the temperature *T* obtained for the chitosan lactate solutions (the CH/LA/β-GP system) and chitosan chloride (the CH/HCL/β-GP system) with β-glycerol phosphate disodium salt pentahydrate (β-GP).

Changes in the values of the storage *G*′ and loss *G*″ moduli as a function of temperature for the systems containing β-GP are shown in Figure 1, and the corresponding changes in the values of the damping factor tan σ are illustrated in Figure 2. In a similar way, the dependence of the complex viscosity η* on the temperature is presented in Figure 3.

In the case of both variants of the solutions with β-GP, the curves of the moduli, shown in Figure 1, can be divided into three characteristic regions corresponding to the successive processes occurring in the samples during the measurement. In each of the distinguished areas, the analyzed biomaterials are characterized by different rheological properties, which describe the current state of their structure and intermolecular interactions.

In the initial stage of the experimental research (region one), the chitosan salt solutions show behaviors characteristic of viscoelastic liquids. Higher values of the loss modulus *G*″ (modulus of the viscous forces) are observed here compared to the values of the storage modulus *G*′ (modulus of elasticity). At the same time, with increasing temperature, a decrease in the values of both moduli and the complex viscosity η* is noted. This confirms that the viscosity of the liquid decreases when temperature increases [34].

In area two (the sol-gel transition region), the increasing temperature brings about a clear increase in the values of the *G*′and *G*″ moduli, which is the consequence of the formation of the cross-linked structure. What is characteristic here is the domination of the elastic response over the viscous response, and the structures created in this region are distinguished by properties that are typical of solids.

In the last stage (region three), the gelation process takes place at a lower speed, which is caused by the high values of the viscosity of the medium, limiting the particle diffusion process.

Based on the values obtained for the *G*′ and *G*″ moduli, the corresponding values of the damping factor tan σ = *G*″/*G*′ for a given temperature were determined. A graphic illustration of the curves of tan σ is shown in Figure 2.

The analysis resulting from the curves of tan σ shows that the gelation temperature of the solutions depends on the type of acid used as a solvent for the solubilization of the biopolymer. In the case of the chitosan lactate solution (the CH/LA/β-GP system), the phase transition took place at 36.0 °C, and for chloride (the CH/HCL/β-GP system), it was 40.5 °C.

The influence of the type of solvent used on the phase transition process is also illustrated in Figure 3, which shows that for the chitosan salt solutions with β-GP, an increasing temperature causes a decrease in the value of the complex viscosity η* until the gelation temperature is reached by the systems in question. After exceeding the sol-gel transition temperature, the η* values of both systems showed a dynamic increase, while the values obtained for the CH/LA/β-GP variant were higher than the CH/HCL/β-GP.

**Figure 4:**
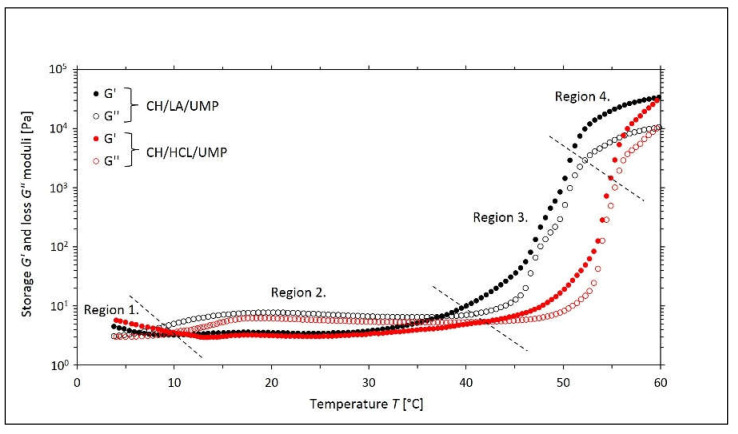
The experimental curves of the changes in the values of the storage *G*′ and loss *G*″moduli as a function of the temperature *T* obtained for the chitosan lactate solutions (the CH/LA/UMP system) and chitosan chloride (the CH/HCL/UMP system) with uridine 5′-monophosphate disodium salt (UMP).

In the case of the solutions prepared with the use of the uridine 5′-monophosphate disodium salt (UMP), the changes in the values of the *G*′ and *G*″ moduli as a function of the temperature obtained during the non-isothermal oscillatory measurements are shown in Figure 4.

Based on the analysis of the course of the above curves, it is possible to distinguish four areas characterizing the processes occurring in the tested samples.

In region one (the highly flexible state), higher values of *G*′ moduli over *G*″ are observed. The systems exhibited typical gel behavior, namely the formation of flexible networks. It is worth bearing in mind that even a slight deformation can damage the resulting internal structure, which is caused by slight differences between the energy values of the heat transfer energy of the macromolecules and the energy of the intermolecular interactions [35].

Area two (the sol region) is characterized by higher values of the loss modulus *G*″ than the storage modulus *G*′. Both chitosan salt solutions exhibited behaviors typical of viscoelastic liquids.

As the temperature increased, the values of both moduli dynamically increased, with the storage modulus *G*′ dominating over the loss modulus *G*″, which proves that the sol-gel transition process started again (region three). By comparing the variants of the tested chitosan salt solutions containing UMP with the systems prepared with the use of β-GP, the difference in the dynamics of changes in the values of the moduli is noticeable. For solutions with UMP, there was a more rapid phase transition compared to samples with β-GP.

In region four (the glassy state), the gelation process was much slower, which is due to the low thermal energy of the macromolecules.

**Figure 5:**
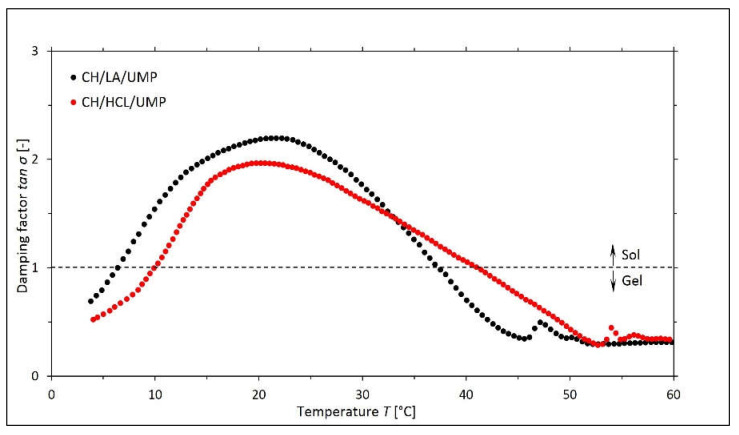
The experimental curves of the changes in the values of the damping factor tan σ *= G*″/*G*′ as a function of the temperature *T* obtained for the chitosan lactate solutions (the CH/LA/UMP system) and chitosan chloride (the CH/HCL/UMP system) with uridine 5′-monophosphate disodium salt (UMP).

In turn, for both chitosan salt solutions with UMP, the curves of the changes in the values of the damping factor tan σ as a function of the temperature are presented in Figure 5.

Based on the above graphs, it can be concluded that for both systems, there are two sol-gel transition points, corresponding to 6.4 °C and 37.3 °C for the CH/LA/UMP system and 10.0 °C and 41.2 °C for the CH/HCL/UMP system. The obtained research results constitute a basis for the assumption that the main driving force in the construction of the spatial structure of the systems with UMP at low temperatures is the hydrogen bonds between the chitosan (CH) skeleton and water molecules, while at high temperatures the formation of the gel occurs because of the hydrophobic interactions between the polymer chains. Similar conclusions were also presented by Li et al. [36], which concerned a solution of chitosan acetate with the addition of dibasic sodium phosphate.

**Figure 6:**
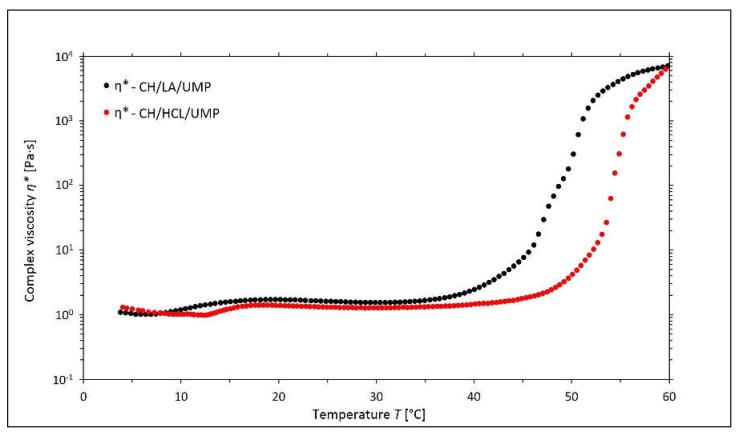
The experimental curves of the changes in the values of the complex viscosity η* as a function of the temperature *T* obtained for the chitosan lactate solutions (the CH/LA/UMP system) and chitosan chloride (the CH/HCL/UMP system) with uridine 5′-monophosphate disodium salt (UMP).

Figure 6 illustrates the curves of the changes in the values of the complex viscosity η* as a function of the temperature.

For the chitosan salt solutions with UMP, in the initial stages of the experiment, there were no significant differences between the values of the complex viscosity. Only after the systems reached a higher gelation temperature (the second sol-gel transition point) was the dynamic increase in the value of η* noticeable. In the final stage of the measurement, the values of the complex viscosity for both samples were equal, but compared to the solutions with β-GP, they were about one order of magnitude higher, which means that the systems containing UMP are potentially more suitable for biomedical applications. A higher viscosity allows the hydrogel to maintain better integrity with the site of tissue damage and prevents flowability.

### 2.2. Nuclear Magnetic Resonance (NMR) Spectra

It has been shown that the NMR technique can be a very sensitive tool to trace any potential variations concerning conformational freedom, which may occur within a molecular system due to (a) phase or structural transitions, (b) changed temperatures/pressure conditions, (c) the presence of spatial confinements, or (d) intermolecular interactions.

The presence of any dynamical hindrance can be exposed either directly with pulse field gradient experiments and diffusion coefficient evaluations [37,38], using spin-lattice relaxation time measurements [39,40] or simply by monitoring NMR spectra evolution. The latter approach appears to be particularly useful in the case of thermoresponsive hydrogel studies [41,42,43], where the volume phase transition (VPT) is clearly manifested as an abrupt drop of the NMR signal amplitude during the deswelling process. This effect can be easily explained considering the significant polymer chain dynamics, which decline over the VPT and consequently decrease the spin-spin relaxation time. Accordingly, there is a substantial fraction of fast decaying FID component build-up, which cannot be detected due to the typically long dead-time of conventional high-resolution NMR probes. Eventually, an apparent NMR signal amplitude drop is observed despite the unchanged population of magnetically active nuclei within the sample. Moreover, the mentioned effect is usually accompanied by NMR line broadening. Therefore, an analysis of NMR signal evolution as a function of the temperature provides an easy and reliable way to detect any stiffening of the molecular system, including the stiffening resulting from the network formation that occurs in hydrogels.

This strategy was employed here to confront the rheological data, which represent macroscopic properties of the studied materials, with the NMR data, which reveal many local interactions occurring at the molecular level. As shown below, the presented results confirm a strong agreement between these two independent methods.

**Figure 7:**
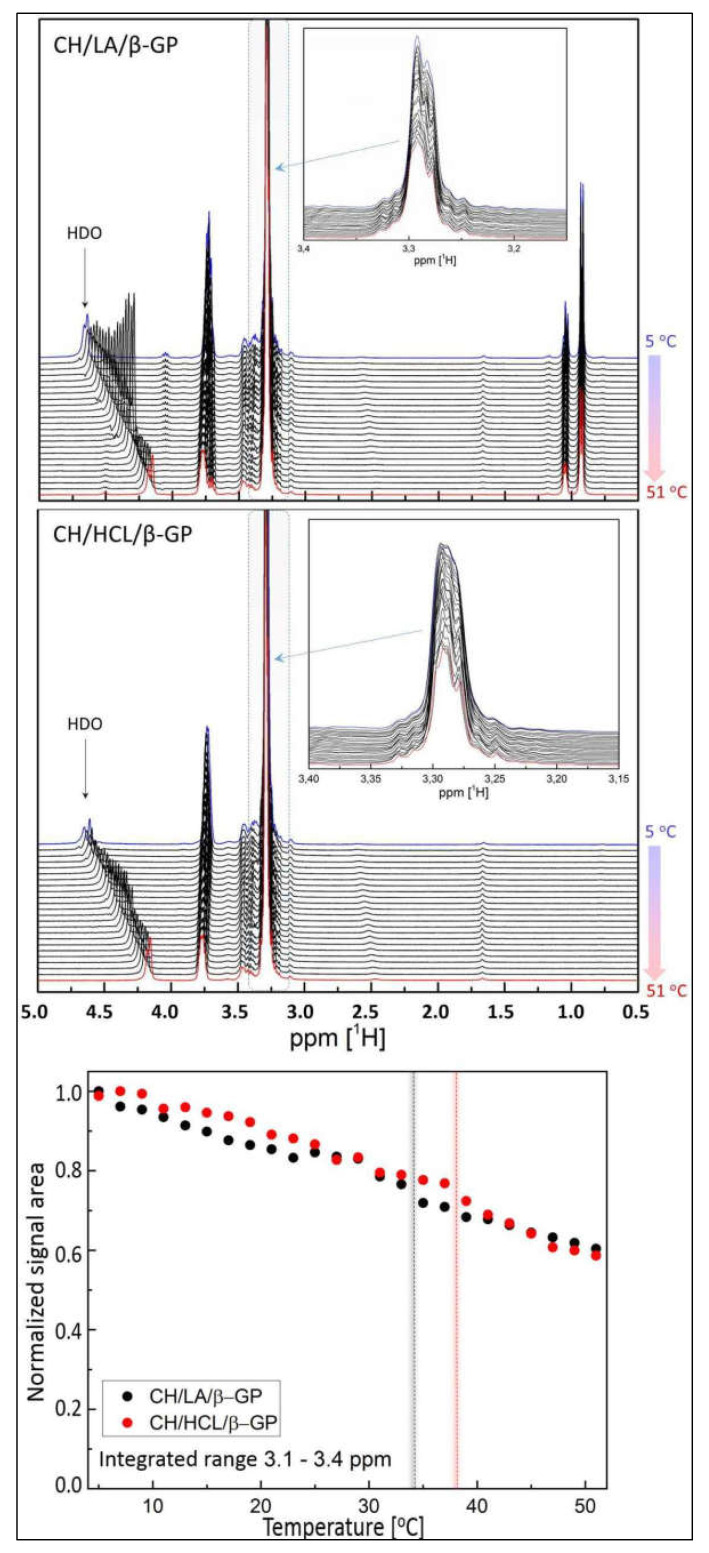
(**top**,**middle**) The ^1^H NMR spectra recorded for the CH/LA/β-GP and CH/HCL/β-GP systems in the temperature range of 5–51 °C; (**bottom**) The NMR signal intensity vs. temperature.

Figure 7 shows the NMR results obtained from the colloidal solutions containing β-GP.

Figure 7 (top, middle) demonstrates the temperature evolution of the ^1^H NMR spectra observed for the CH/LA/β-GP and CH/HCL/β-GP systems. The magnified chemical shift range displayed in the insets reveals the NMR signals attributed to the glucosamine ring [44,45]. The spectrum of the CH/LA/β-GP system shows additional signals in the low ppm range, which are characteristic of lactic acid. It is also worth mentioning that the HDO solvent signal position is temperature sensitive, and its shielding exhibits a nearly linear temperature dependence (in the temperature range studied) [46], which is clearly visible in all NMR spectra presented.

Figure 7 (bottom) presents the normalized NMR signal area as a function of the temperature, analyzed in the chemical shift range typical for protons located at the glucosamine ring. The data show gradual reductions of the NMR peak area throughout the whole temperature range measured and do not exhibit any evident variations at specific temperatures, which signify a drastic change in polymer chain mobility. Nevertheless, in both cases, it is possible to estimate the temperature (marked with vertical black and red dashed lines) where the amplitude variation step is slightly more pronounced and could be related to the sol-gel transition. In the case of the CH/LA/β-GP system, the drop takes place between 33 °C and 35 °C, while in the case of the CH/HCL/β-GP system, a similar drop is shifted towards the higher temperatures and appears between 37 °C and 39 °C.

Interestingly, these subtle transitions observed in the NMR spectra evolution corroborate the well-presented rheological data above. Both the storage and the loss of moduli data (Figure 1), together with the damping factor data (Figure 2), indicate that there is approximately a 4 °C difference concerning the sol-gel transition between the two samples, which agrees with NMR results.

**Figure 8:**
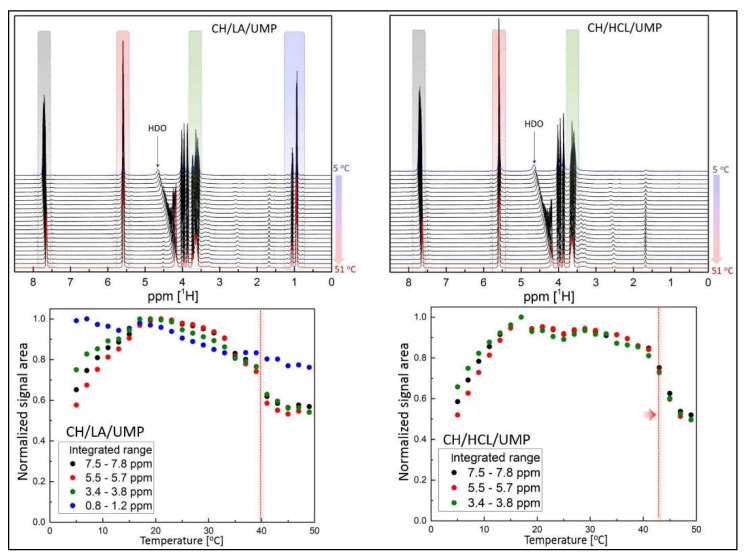
(**top**) The ^1^H NMR spectra recorded for the CH/LA/UMP and CH/HCL/UMP systems in the temperature range of 5–51 °C; (**bottom**) The NMR signal intensity vs. temperature.

In turn, Figure 8 (top) shows the ^1^H NMR spectra acquired for the CH/LA/UMP and CH/HCL/UMP samples under different temperature conditions. There are additional peaks visible at ~5.6 ppm (marked in red) and ~7.6 ppm (marked in gray), which indicate the presence of UMP [47]. The CH/LA/UMP spectrum reveals the extra NMR peaks in the low ppm range (marked in blue), confirming the presence of lactic acid. The chemical shift range marked in green represents the signals attributed to the glucosamine ring. The solvent signal position varied with the temperature in the same way as observed above.

Figure 8 (bottom) illustrates the NMR signal area as a function of the temperature for the CH/LA/UMP and CH/HCL/UMP systems, respectively. The color of the dots corresponds to the colors of the integrated chemical shift ranges displayed in NMR spectra. Interestingly, the presented data differs significantly from the data observed in Figure 7. Instead of monotonously decreasing the NMR signal intensity, one can recognize three distinct temperature ranges here, i.e., (a) a low-temperature range where the NMR signal amplitude drops substantially as the temperature decreases, (b) an intermediate temperature range where the NMR signal amplitude remains relatively stable and forms a plateau, (c) a high-temperature range where the NMR signal amplitude decreases again quite rapidly with increasing temperatures. Such a result suggests that there are two temperature points beyond this, where the system undergoes stiffening.

This finding very much agrees with the rheological data presented in Figure 4 and Figure 5, where the storage and loss moduli together with the damping factor are shown, respectively, and indicate two sol-gel transitions, one occurring at a lower temperature and the second at a higher temperature. Moreover, it is worth emphasizing that the NMR signal amplitude vs. temperature relationship observed in the CH/LA/UMP and CH/HCL/UMP systems reveal sudden and distinct changes at specific temperatures (marked with a red dotted line), which seems to be a manifestation of the sol-gel transition. Furthermore, in the case of the CH/HCL/UMP sample, one can observe that this substantial NMR amplitude drop is shifted towards a higher temperature with respect to the corresponding drop occurring in the case of the CH/LA/UMP system. The shift is approximately 4 °C and correlates nicely to the sol-gel transition shift observed via rheology.

## 3. Conclusions

Based on the measurements, it was found that the type of cross-linking agent used significantly influenced the course of the sol-gel transition of the colloidal chitosan salt solutions.

The phase transition of the systems containing β-glycerol phosphate disodium salt pentahydrate (β-GP) during heating with a constant temperature rising rate occurs in three distinctive regions: (1) the solutions show behavior typical of viscoelastic liquids, (2) the sol-gel transition area, and (3) the slow gelation process at high temperatures. On the other hand, in the case of the chitosan solutions with uridine 5′-monophosphate disodium salt (UMP), four areas describing the course of the sol-gel transition were identified: (1) the highly flexible state, (2) the sol area, (3) the re-phase transition region, and (4) the glassy state. Thus, for the solutions containing β-GP (the CH/LA/β-GP and CH/HCL/β-GP systems), there was one sol-gel transition point at 36.0 °C and 40.5 °C, respectively. However, the samples with UMP had two gelation points: 6.4 °C and 37.3 °C for the CH/LA/UMP system and 10.0 °C and 41.2 °C for the CH/HCL/UMP system.

Considering the potential use of chitosan systems as injectable thermosensitive hydrogels in tissue engineering, solutions consisting of chitosan lactate seem more appropriate. The phase transition temperatures of these systems are closer to the physiological temperature of the human body compared to the solutions obtained from chitosan chloride.

The observations from the rheological studies have been fully confirmed with the results of the experiments carried out with nuclear magnetic resonance spectroscopy, which provided information on the interactions occurring in the tested samples at the molecular level. Thus, the use of both measurement techniques ensures a comprehensive review of the behaviors of the chitosan solutions sensitive to temperature changes, which is particularly important when designing hydrogel matrices for biomedical use.

The mechanism of chitosan gel formation was first proposed by Chenite et al. [48] and Filion et al. [49]. Chenite et al. justified the formation of gels with electrostatic interactions, while the second research group considered that the reduction of positively charged amino groups occurs, which is caused by a decrease in chitosan pKa. The released H^+^ protons are captured by the GP^2−^ groups. The verification of the above proposals was created based on structural and rheological tests. Studies with the use of β-GP are presented in publications [33,34,35,50,51] and with the use of UMP in the articles [32,33]. On the other hand, Peters et al. [52] demonstrated the molecular structure containing a ribose sugar with purines or pyrimidines that can be formed with intermolecular attractions, including π-π stacking, electrostatic interactions and hydrogen bonds. Interesting studies were also conducted by Grinberg et al. [53]. The researchers analyzed the thermoresponsivity of systems containing chitosan and β-GP by high-sensitivity differential scanning calorimetry.

## 4. Materials and Methods

### 4.1. Materials and Preparation of Chitosan Salt Solutions

Chitosan (CH)—product no. 50494-100GF (degree of acetylation (DA)= 18.2%, weight average molecular mass (M_w_)= 680 kg·mol^−1^, number average molecular mass (M_n_)= 110 kg·mol^−1^, polydispersity index (PDI)= 6.18), two types of acids: lactic acid (LA)—product no. L6661-100ML and hydrochloric acid (HCL)—product no. H1758-100ML, and cross-linking agents: β-glycerol phosphate disodium salt pentahydrate (β-GP)—product no. 50020-100G and uridine 5′-monophosphate disodium salt (UMP)—product no. U6375-10G were used in the preparation of solutions. All analytical grade reagents were supplied by Sigma-Aldrich (Poznan, Poland).

Chitosan salt solutions (2.5% *w*/*v*) were prepared by dissolving 0.4 g of CH in 16 mL of 0.1 mol/L LA or HCL. After thoroughly mixing, the samples were left at room temperature for 24 h to allow the biopolymer to dissolve completely. The next step was to prepare the cross-linking agent solutions. For this purpose, 2 g of β-GP was dissolved in 2 mL of deionized water at 4 °C, or 2 g of UMP was dissolved in 2.5 mL of deionized water, and these solutions were then added gradually to the chitosan salt solutions and were stirred at the same time. The samples with β-GP were stored at 4 °C for about 2 h, and the solutions with UMP were left at room temperature.

### 4.2. Rheological Studies

Rheological measurements were performed using the Anton Paar Physica MCR 301 rotational rheometer (Anton Paar, Warsaw, Poland) equipped with a cone-plate measuring system (diameter—50 mm, angle—1°, and truncation—0.048 mm). The sol-gel transition temperatures were determined based on oscillatory tests at a constant deformation value (angular frequency ω = 5 s^−1^ and strain amplitude $\dot{\gamma }\;$= 1%). The gelation processes were carried out under non-isothermal conditions, maintaining a constant heating rate of 1 °C·min^−1^. The solutions with β-GP were tested from 4 °C (sample storage temperature) to 60 °C, while for the systems with UMP, the samples were first cooled from room temperature (storage conditions) to 4 °C and then heated up to 60 °C.

### 4.3. NMR Experiments

NMR spectra were acquired using the Agilent NMR 400 MHz spectrometer equipped with a double channel X{^1^H} OneNMR probe. A simple one-pulse excitation experiment with dipolar decoupling was applied to record the proton spectra. The NMR probe temperature was monitored and controlled using a variable temperature system with ~0.1 °C accuracy. Samples were solved in D_2_O and placed in 5 mm NMR tubes. In the case of the samples containing β-GP, the first spectrum was recorded at 5 °C, and subsequently, the probe temperature was increased at 2 °C intervals. In the case of the samples with UMP, two individual sets of experiments were performed, i.e., initially, the first sample was monitored from 15 °C up to 51 °C and then a separate second sample was measured from 15 °C down to 5 °C. The temperature interval was 2 °C.

## 5. Patents

Majsterek I., Modrzejewska Z., Pieklarz K., Tylman M.; Method for producing chitosan gels forming in the human body temperature, intended for injection scaffolds for breeding of nerve cells. Lodz University of Technology, Lodz. Poland. Patent application 235369. Publ. 29.06.2020 WUP.

## Data Availability

Data sharing is not applicable to this article.
